# Risk factors for glaucoma are reflected in abnormal responses to frequency-doubling technology screening in both normal and glaucoma eyes

**DOI:** 10.1038/s41598-022-15891-3

**Published:** 2022-07-09

**Authors:** Aiko Iwase, Tae Tsutsumi, Makoto Fujii, Shoichi Sawaguchi, Makoto Araie

**Affiliations:** 1 Tajimi Iwase Eye Clinic, 3-101-1 Honmachi, Tajimi, Gifu Prefecture 507-0033 Japan; 2 Department of Ophthalmology, University of Tokyo Graduate School of Medicine, Tokyo, Japan; 3 Division of Health and Sciences, Osaka University Graduate School of Medicine, Osaka, Japan; 4 Department of Ophthalmology, Faculty of Medicine, University of the Ryukyu, Okinawa, Japan; 5 Kanto Central Hospital of the Mutual Aid Association of Public School Teachers, Tokyo, Japan

**Keywords:** Eye diseases, Optic nerve diseases

## Abstract

The frequency-doubling technology (FDT) screening test (FDT-C-20-1) has adopted in many recent population-based glaucoma surveys, but factors associated with false-positive (FP) responses to FDT-C-20-1 in normal eyes and false-negative (FN) responses in glaucoma eyes were not known. These factors were investigated in a population-based setting using the data from 3805 normal eyes (2381 subjects) and 272 eyes with definite glaucoma (215 subjects) in the Kumejima Study participants with reliable FDT-C-20-1 results. Considering the presence of at least one abnormal test point (*P* < 0.01) as abnormal, the specificity and sensitivity of FDT-C-20-1 for glaucoma were 91.8% (95% confidence interval, 91.1 ~ 92.5) and 56.3% (47.0 ~ 62.5), respectively. Multivariate linear mixed-model logistic regression analysis showed correlations with older age, worse visual acuity, greater β-peripapillary area (*P* < 0.001 for all comparisons) and more myopic refraction (*P* = 0.030) with the FP responses in normal eyes, and normal-tension glaucoma (*P* = 0.043), a better mean deviation value of Humphrey perimetry (*P* = 0.001), larger rim area (*P* = 0.041), and absence of disc hemorrhage (*P* = 0.015) with the FN responses in glaucoma eyes. In a population-based setting, abnormal responses to FDT-C-20-1 indicate the presence of a risk factor for glaucoma in normal eyes and risk factors for more rapid progression in glaucoma eyes.

## Introduction

The global prevalence of glaucoma, a leading cause of blindness worldwide, for people aged 40 to 80 years is about 3.5%, and the global number of cases of glaucoma is expected to increase to approximately 112 million due to the rapidly increasing aging populations^1^. Since a glaucoma diagnosis is established when structural changes in the optic nerve head and/or retina that are considered to be glaucomatous match the location of the visual field defects (VFDs), their identification is indispensable in population- or community-based screening for glaucoma^2^. Frequency-doubling technology (FDT) perimeters are relatively easy to operate and FDT screening programs require only about 1 to 1.5 min of subject cooperation^3–5^. A systematic review and meta-analysis suggested that FDT screening for glaucoma was one of the best single methods in the diagnostic performance^6^. In fact, FDT screening programs have been adopted in many recent population-based glaucoma surveys^7–17^ and community-based studies^18–20^ to screen for the VFDs caused by glaucoma; some of the recent population-based studies have adopted the FDT screening program as the only test method to screen for VFDs^10,14,17^. However, more than a few population- or community-based studies^8,21–24^ have reported relatively high false-positive (FP) and false-negative (FN) response rates associated with the FDT test results when screening for glaucoma. A previous study reported that unreliable or unfeasible FDT test results occurred more frequently in patients with glaucoma than in healthy subjects, suggesting that unreliable or unfeasible FDT test results still contain clinically useful information^25^. Thus, the factors associated with abnormal or positive responses (FP-responses) to the FDT screening programs in normal eyes and those of normal or negative responses (FN-responses) in glaucoma eyes, if they exist, also may provide clinically useful information. However, to the best of our knowledge, no previous studies have addressed this problem in the general population in which application of FDT seems most clinically relevant.

The Kumejima Study is a population-based epidemiologic study of 3762 participants that focused on ocular diseases in Kumejima in southwest Japan^26,27^, where all participants underwent the FDT screening program and glaucoma was diagnosed based on the stereo fundus photographs and the results of the Humphrey Field Analyzer Central 24-2 Swedish interactive threshold algorithm (SITA) standard program (HFA 24-2, Carl Zeiss Meditec, Dublin, CA). Using eyes that were confirmed to be ophthalmologically normal and those with definite glaucoma identified in this population-based study, we attempted to find clinically useful factors related to FP responses to the FDT screening test in normal eyes and to FN responses in eyes with primary glaucoma.

## Results

No participants reported history of refractive surgery. Among In 3805 ophthalmologically normal eyes (2381 subjects) and 272 eyes (215 subjects) with definite glaucoma with reliable FDT results, the FDT C-20-1 screening program detected no abnormal test point with *P* < 0.01^28^ in 3492 ophthalmologically normal eyes and detected at least one abnormal test point in 153 eyes with definite glaucoma, yielding a specificity of 91.8% (95% confidence interval, 91.1 ~ 92.5%) and a sensitivity of 56.3% (95% confidence interval, 47.0 ~ 62.5%) for screening eyes with definite glaucoma (Table 1).

**Table 1:** Sensitivity and specificity in glaucoma and normal eyes.

	≥ 1 abnormal points	
	Yes	No
Definitive glaucoma (272 eyes)	153	119
Normal eyes (3805 eyes)	313	3492
Sensitivity, 56.3% (95% confidence interval, 47.0–62.5%)		
Specificity, 91.8% (95% confidence interval, 91.1–92.5%)		

It was confirmed that the explanatory variables with *P* values of ≤ 0.10 in the univariate analysis that were included in the multivariate analysis were not strongly intercorrelated (Pearson’s correlation coefficient − 0.60 << 0.60). Generalized linear mixed-modeled multiple logistic regression analysis showed that older age (*P* < 0.001), worse VA (*P* < 0.001), left laterality (*P* = 0.039), more myopic refraction (*P* = 0.030), shallower anterior chamber depth (ACD) (*P* = 0.043) and larger β-peripapillary atrophy (PPA) area (*P* < 0.001) were correlated significantly with abnormal or FP responses to the FDT C-20-1 screening program in normal eyes, while POAG with normal pressure (normal-tension glaucoma) (*P* = 0.043), right laterality (*P* = 0.007), greater rim area (*P* = 0.041), better MD value (*P* = 0.001), and absence of disc hemorrhage (*P* = 0.015) were correlated significantly with negative, but incorrect or FN responses in glaucomatous eyes (Tables 2,3).

**Table 2:** Results of univariate and multivariate logistic regression analysis in 3638 normal eyes of 2255 normal subjects for whom all parameter values for analysis were available.

	≥ 1 Abnormal test points on FDT C-20-1 screening program		*P* value*	Results of multivariate logistic regression analysis (generalized linear mixed model)		
	No	Yes		Estimate^†^	95% Confidence Interval	*P* Value
No. eyes	3354	284				
Age (years)	56.1 (11.7)	66.5 (12.5)	0.001	0.082	0.050 – 0.115	< 0.001
Male/female	1722/1632	118/166	0.492			
Presence/absence of diabetes	244/3110	31/253	0.672			
Best-corrected visual acuity (logMAR)	− 0.04 (0.10)	0.05 (0.17)	0.001	5.11	2.66 – 7.57	< 0.001
Right/left	1801/1553	144/140	0.037	− 0.395	− 0.771 to − 0.020	0.039
Intraocular pressure (mmHg)	14.8 (2.9)	14.3 (3.3)	0.199			
Central corneal thickness (μm)	516 (33)	515 (38)	0.481			
Spherical equivalent refraction (diopters)	0.01 (1.67)	0.41 (1.88)	0.070	− 0.22	− 0.43 to − 0.02	0.030
Axial length (mm)	23.45 (0.90)	23.24 (0.88)	0.007	− 0.22	− 0.63 to 0.18	0.277
Anterior chamber depth (mm)	3.15 (0.37)	2.92 (0.36)	< 0.001	− 0.96	− 1.89 to − 0.03	0.043
Disc area (mm^2^)	2.54 (0.50)	2.54 (0.50)	0.936			
Rim area (mm^2^)	1.67 (0.30)	1.61 (0.33)	0.674			
Vertical cup/disc ratio	0.55 (0.09)	0.58 (0.10)	0.136			
β-peripapillary atrophy area (mm^2^)	0.40 (0.57)	0.84 (1.01)	0.003	0.81	0.44–1.17	< 0.001
Presence/absence of disc hemorrhage	5/3349	1/283	0.110			

The data are expressed as the mean (standard deviation).

*logMAR* logarithm of the minimum angle of resolution, *FDT* frequency-doubling technology.

*Univariate logistic regression analysis (generalized linear mixed model).

^†^Positive values of estimates indicate false-positive or abnormal responses to the FDT screening test in normal eyes.

**Table 3:** Results of univariate and multivariate logistic regression analysis in 153 eyes with definite glaucoma of 126 patients with glaucoma for whom all parameter values used for analysis were available.

	≥ 1 Abnormal test points on FDT C-20-1 screening program		*P* value*	Results of multivariate logistic regression analysis (generalized linear mixed model)		
	No	Yes		Estimate^†^	95% confidence interval	*P* Value
No. eyes	73	80				
Age (years)	66.4 (12.1)	68.7 (11.3)	0.240			
Male/female	39/34	45/35	0.700			
Presence/absence of diabetes	7/66	9/71	0.773			
Best-corrected visual acuity (logMAR)	0.0 (0.14)	0.05 (0.22)	0.135			
Right/left	51/22	36/44	0.014	− 2.05	− 3.49 to − 0.61	0.007
Intraocular pressure (mmHg)	15.8 (2.6)	17.1 (3.8)	0.044	0.095	− 0.159 to 0.349	0.447
Central corneal thickness (μm)	520 (35)	510 (33)	0.103			
Spherical equivalent refraction (diopters)	0.24 (1.85)	0.24 (2.04)	0.958			
Axial length (mm)	23.38 (0.88)	23.40 (0.83)	0.765			
Anterior chamber depth (mm)	2.99 (0.40)	2.97 (0.40)	0.947			
Disc area (mm^2^)	2.83 (0.54)	2.68 (0.58)	0.141			
Rim area (mm^2^)	1.42 (0.28)	1.14 (0.33)	0.007	− 3.56	− 6.955 to − 0.155	0.041
Vertical cup/disc ratio^†^	0.69 (0.09)	0.77 (0.10)	0.004	− 1.28	− 11.32 to 8.77	0.794
β-peripapillary atrophy area (mm^2^)	0.81 (0.10)	1.07 (0.97)	0.104			
Presence/absence of disc hemorrhage	3/70	10/70	0.088	− 4.68	− 8.37 to − 0.99	0.015
Mean deviation (decibels)	− 2.70 (3.59)	− 8.72 (7.07)	< 0.001	− 0.36	− 0.56 to − 0.16	0.001
High-tension group/normal tension group	24/49	49/31	0.006	− 1.86	− 3.66 to − 0.06	0.043
POAG/PACG	53/20	54/26	0.533			

The data are expressed as the mean (deviation). The high-tension group indicates eyes with primary angle-closure glaucoma and primary open-angle glaucoma with elevated pressure (> 21 mmHg); the normal-tension group are those with primary open-angle glaucoma with normal pressure (≤ 21 mmHg).

*logMAR* logarithm of the minimum angle of resolution, *FDT* frequency-doubling technology, *dB* decibels, *POAG* primary open angle glaucoma, *PACG* primary angle closure glaucoma.

*Univariate logistic regression analysis (generalized linear mixed model). Negative values of estimates indicate incorrect or false-negative results in the FDT test in glaucomatous eyes.

## Discussion

The subjects of the current study had ophthalmologically normal eyes and those with definite glaucoma were identified in the Kumejima Study^26,27^. The FDT-C-20-1 screening test was completed in both eyes in 3487 out of 3572 who underwent the test and in one eye in 51 of those, yielding reliable results in 2227 examinees in both eyes and in 1045 examinees in one eye. These figures compared favorably with those reported in community- or population-based surveys in other ethnic groups^22,24^. The sensitivity for detecting glaucomatous eyes, 56.3%, and the specificity in ophthalmologically normal eyes, 91.8%, agreed well with those found in the Tajimi Study, i.e., 55.6% and 92.7%, respectively^8^. Since the prevalence of glaucoma in Japanese individuals aged 40 years and older is thought to be about 6%^7,26,27,29^, the FDT-C-20-1 screening test applied in 1000 Japanese individuals in the general population would yield 109 persons with abnormal test results (60 × 0.56 = 34 + 940 × 0.08 = 75), which means that about 69% (75/109) of persons with abnormal FDT test results would be normal (FP responses), while 43% (26/60) of patients with glaucoma would be overlooked (FN responses). Thus, information regarding factors associated with FP or FN responses to the FDT test is clinically relevant.

The current study showed that in otherwise normal eyes, older age, worse VA, larger β-PPA area, left laterality, more myopic refraction (more negative SE) and shallower ACD were associated significantly with abnormal or FP responses on the FDT screening test after adjustment for the confounding effects of other factors. It seems understandable that older age or worse VA is more likely to be associated with FP responses to the FDT screening test in normal eyes, but the reasons why otherwise normal eyes with relatively greater β-PPA area were more likely to yield FP responses to the FDT screening test may need discussion. Since a larger β-PPA area is a risk factor for glaucoma development and/or progression according to a previous review study^30^, these eyes might be more likely to have subclinical functional weaknesses, suggesting that otherwise normal eyes with FP responses to the FDT screening test may have a relative risk for developing POAG in the future. In normal Japanese eyes, myopic eyes are more likely to have larger β-PPA area^31^, and more likely to show slight depression in the Humphrey Perimeter-measured visual field sensitivities^32^. Lee et al.^33^ recently reported that non-glaucoma eyes with moderate myopia with growing β-PPA area are more likely to show progression of temporal non-glaucomatous VF defects. Taken together, it does not seem unreasonable to assume that non-glaucomatous normal eyes with larger β-PPA areas are more likely to have some subclinical functional damage. A significant association of FP responses to the FDT test with more myopic refraction in the current normal eyes is also compatible with the above speculation. The average SE of the current normal eyes was emmetropic, indicating that this group included both myopic and hyperopic eyes and average β-PPA area in this group was 0.4 mm^2^. Additionally, normal eyes with shallower ACD are more likely to yield FP responses to the FDT-test. Since shallower ACD is not associated with myopia, this finding is not compatible with the other current findings that normal eyes with larger β-PPA area or more myopic refraction are more likely to yield FP responses to the FDT test. In the current normal eyes, ACD and age showed a moderate negative correlation (Pearson’s correlation coefficient = − 0.555), that is, older the age, shallower the ACD. Since older age is a definite factor associating with FP responses to the FDT test in normal eyes, it may be possible that effects of ACD on the FDT performance was somewhat overestimated in the current subject eyes which are more likely to have both shallower ACD and older age. Elucidation of the effects of ACD on the FDT performance seems to need future studies in a population with less inter-correlation between age and ACD. On the other hand, it seems less likely that effects of myopic refraction on FP responses to the FDT test was overestimated in the current subject eyes, since SE and age showed moderate positive correlation (r = 0.485), that is, younger age is associated with more myopic refraction. In the current normal eyes, left eyes were more likely to yield abnormal or FP responses to the FDT screening test, which agreed with the results reported by Tatemichi et al.^34^. Since the right eye was examined first both in their study and the current study, this finding may suggest an order effect between the two eyes.

In eyes with glaucoma, right laterality, a better MD value, greater rim area, absence of disc hemorrhage, and POAG with normal pressure or normal tension glaucoma were associated significantly with FN or normal responses to the FDT screening test after adjustment for the confounding effects of other factors. In other words, a worse MD value, smaller rim area, presence of disc hemorrhage, POAG with elevated pressure and PACG, and the left eye were independent factor associated with detection of glaucomatous eyes using the FDT screening test. The association of left laterality with abnormal or positive responses to the FDT screening test in glaucoma eyes agreed with the finding obtained in normal eyes currently and previously found^34^. It seems reasonable that glaucoma eyes with a worse MD value and smaller rim area, i.e., those with more damage, were more likely to be detected using the FDT screening test as previously reported^8^. The presence of a disc hemorrhage in glaucoma eyes is a well-known risk factor for further glaucoma progression^30^. Murata et al. compared the POAG with elevated pressure and that with normal pressure with matched Heidelberg Retina Tomography-determined optic disc appearance and standard automated perimetry (SAP) results and reported that the FDT test yielded significantly more abnormal test points in the POAG group with elevated pressure than in that with normal pressure^35^. Kogure et al. reported more abnormal points with FDT than with SAP in POAG with elevated pressure, while the number of abnormal points was equivalent between FDT and SAP in POAG with normal pressure^36^. Although not always confirmed, functional abnormalities in the magnocellular pathway were more sensitively reflected by FDT perimetry^37^, and axonal transport to the magnocellular layers of the lateral geniculate body was decreased more notably than that to the parvocellular layer in a monkey glaucoma model with chronically elevated intraocular pressure (IOP)^38^. The current results seem to agree with these previous results. The current result that the FDT screening test was more likely to detect POAG with elevated pressure and PACG than normal-tension glaucoma has clinical implications, since the rate of progression of untreated POAG with elevated pressure is generally higher than that of untreated normal-tension glaucoma^39^, and PACG, which has a high prevalence in Asia^40,41^, is more likely to result in blindness than POAG^42,43^. Combined with the presence of disc hemorrhage as an independent factor contributing to detection of glaucoma eyes using the FDT screening test, the current results implied that the FDT screening test has potential to detect glaucomatous eyes that are more likely to show more rapid progression and, thus, to be candidates for immediate care.

A limitation of the current study was the relatively small number of patients with POAG or PACG, about 55% (126/233) of all patients with POAG or PACG found in the Kumejima study^26,27^, mainly because we needed reliable fundus planimetric results as explanatory variables for final analysis and thus excluded eyes without good-quality stereo fundus photograph or those that had undergone cataract surgery. Practically, a subject from whom acceptable fundus photographs could not be obtained in screening examination would be usually advised to undergo definitive examination, and glaucoma, if it exists, would be less likely to be overlooked. Another limitation was that the exclusion of eyes with secondary glaucoma from the current final analysis might have made the results less clinically useful. However, during screening, eyes with secondary glaucoma were detected more easily because of the presence of coexisting eye diseases or their past history. Finally, it must discussed why significant factors associated to abnormal responses to the FDT screening test were somewhat different between the current normal and glaucoma eyes. It seems understandable that factors directly relating to glaucomatous damage such as rim area or disc hemorrhage were more likely to be detected in glaucoma eyes. On the other hand, age, best-corrected visual acuity and β-PPA area which showed highly significant association (*P* < 0.001) in the current normal eyes could not be detected as significant factors in the current glaucoma eyes. These factors showed highly significant differences between the current normal and glaucoma eyes (*P* ≤ 0.002 after correction for multiple comparison). Further, much smaller number of the current glaucoma eyes than normal eyes might reduce the statistical power to detect possible association in the glaucoma eyes. Thus, these points, especially, differences in demographics between the current normal and glaucoma eyes should be taken into consideration in interpreting the current result.

In summary, the results of the FDT C-20-1 screening test in the Kumejima Study participants indicated that the abnormal responses to this test cannot only detect glaucoma eyes but also contain clinically useful information. Normal eyes with abnormal or FP responses were more likely to be associated with risk factors for glaucoma, and glaucoma eyes with abnormal responses detected by this test were significantly more likely to show rapid progression.

## Methods

### Subjects

The Kumejima Study conformed to the tenets of the Declaration of Helsinki and regional regulations; the ethics committee of the local government & health department (Kumejima Town) approved the study protocol. All participants provided written informed consent before the examinations. The study was conducted between May 2005 and August 2006 in Kumejima, a southwest island in Okinawa Prefecture, Japan. All residents aged 40 years or older were encouraged to participate and after excluding residents who had died, moved, or could not be located during the study period, 4632 residents were eligible for the study^26,27^.

Since we reported the details of the examinations and diagnoses in our previous studies^26,27^, they are summarized briefly here. The screening examination consisted of a structured interview; measurements of body weight, height, and systemic blood pressure; and ocular examinations performed by experienced ophthalmologists and examiners. The ophthalmic examinations included measurement of the uncorrected and BCVA, refraction, IOP, central corneal thickness (CCT), anterior chamber depth (ACD), axial length (AL), slit-lamp examination, gonioscopy, ophthalmoscopy, fundus photography, and VF testing. A pair of sequential stereoscopic optic nerve head photographs at a parallax of about 8 degrees and plain fundus photographs was obtained using a digital nonmydriatic fundus camera (TRC-NW7, Topcon, Tokyo, Japan) in both eyes of the subjects. The IOP was measured three times using Goldmann applanation tonometry and the median value was recorded; the CCT was determined by specular microscopy (SP-2000, Topcon); and the central ACD and AL were determined using the IOLMaster (Carl Zeiss Meditec). The peripheral ACD was scored according to the van Herick method and the gonioscopic findings according to Shaffer’s grading system using a Goldmann two-mirror lens. The VF was examined using FDT perimetry in the right eye first with the C-20-1 screening program (Carl Zeiss Meditec). Participants were referred for a definitive examination if they were suspected of having ocular abnormalities including glaucoma after meeting one or more of the following criteria during the screening examination: corrected VA below 20/30, IOP over 19 mmHg, vertical cup/disc (vC/D) ratio of 0.6 or more, superior (11–1 o’clock hours) or inferior (5–7 o’clock hours) rim width/disc diameter of 0.2 or less, bilateral asymmetry of the vC/D ratio of 0.2 or more, nerve fiber layer defects or splinter disc hemorrhages, abnormal findings on slit-lamp examination or fundus photographs, van Herick grade of 2 or less, and at least one abnormal test point with *P* < 0.01 in the C-20-1 test results of the FDT VF testing. The definitive examination included detailed gonioscopy and VF testing with the HFA 24–2 program before pupillary dilatation and detailed slit-lamp biomicroscopy and fundus examinations after pupillary dilation. Glaucoma was diagnosed based on the clinical records obtained during all examinations; evaluations of slit-lamp and gonioscopic findings; disc, retinal nerve fiber layer, and VFs^26,27^; and the International Society of Geographic and Epidemiologic Ophthalmology criteria^2^.

### Planimetry on stereoscopic fundus photographs

Since the current analyses also included the planimetric fundus results as covariates, the current planimetric method is summarized briefly here^44,45^. An experienced ophthalmologist (T.T.) re-examined all stereo photographs. While stereoscopically viewing the optic disc, the disc contour, defined as the inner boundary of the peripapillary scleral ring, was determined by a series of seven points with spline interpolation, and the cup contour, defined as the point of change of slope from the cup wall to the neural rim, was determined as a closed curve by an unlimited number of points placed on the computer monitor using a computer mouse. The β-PPA area was characterized by visible sclera and large choroidal vessels owing to the absence of the retinal pigment epithelium and also determined as a closed curve by an unlimited number of points placed on the outer boundary of the β-zone and that of the peripapillary scleral ring on the computer monitor. The fovea also was determined and the disc center calculated automatically as the center of gravity of the disc area. After correcting for magnification by the corneal curvature, AL, and refractive error according to the formula provided by the manufacturer, the planimetric parameters, disc, rim, cup, and β-PPA areas in mm^2^ were calculated automatically and the reproducibility of the measurement results were reported previously^44,45^.

### Inclusion and exclusion of subject eyes

In the Kumejima Study, 3762 (participation rate, 81.2%) of the 4632 eligible residents aged 40 years or older underwent a screening examination and 2399 were referred for definitive examinations; 185 refused, declined, or could not participate. As a result, 2214 subjects underwent a definitive examination including VF testing using the Humphrey Field Analyzer 24–2 SITA (HFA 24–2) test program. Of the 3572 subjects who visited the screening examination site, the FDT results were obtained in both eyes of 3487 subjects and 25 and 26 right and left eyes, respectively, of 25 and 26 subjects, but not in both eyes of 34 subjects, because of the participants’ inability to understand the method, fatigue, lack of cooperation, or best-corrected visual acuity (BCVA) below 20/200. Considering FDT results with fixation losses of one-third or more and/or FP errors of one-third or more as unreliable^20^, reliable FDT results were obtained in both eyes of 2226 subjects and 701 right eyes of 701 subjects and 344 left eyes of 344 subjects. After excluding 1692 eyes with definite glaucoma or glaucoma suspects, and those with any retinal diseases, ocular trauma, brain diseases, BCVA below 0.5, or high myopia exceeding -8 diopters, 3805 normal eyes (2381 subjects) had reliable FDT results. Among 363 eyes with definite glaucoma (270 subjects), 272 eyes (215 subjects) had reliable FDT results. Specificity for screening normal eyes and sensitivity for screening eyes with definite glaucoma were determined in these 3805 normal eyes (2381 subjects) and 272 eyes (215 subjects) with definite glaucoma.

**Figure 1:**
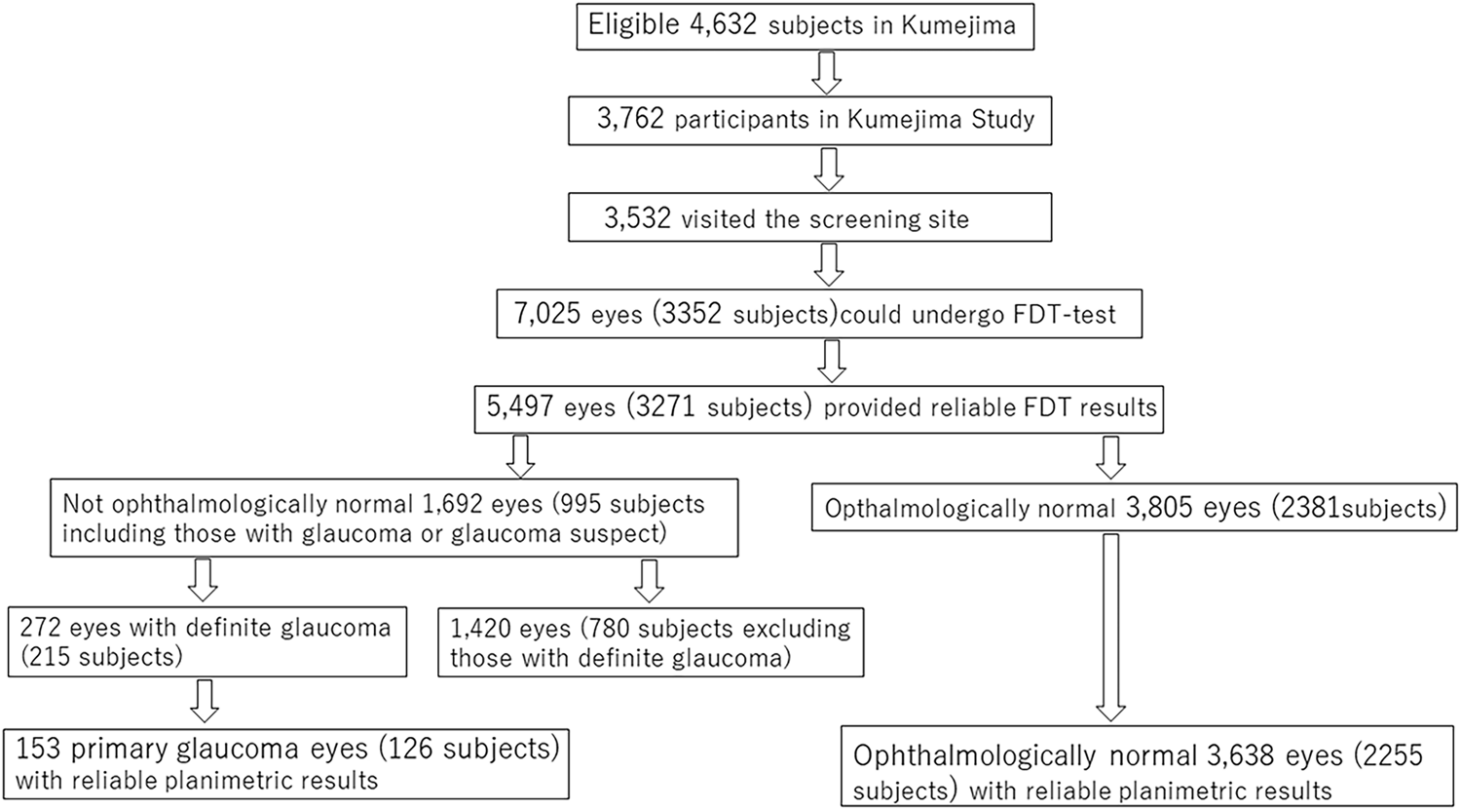
The process of determining the target eye of this study in the Kumejima Study is shown. Finally, 3638 normal eyes of 2255 subjects and 153 eyes with primary open-angle glaucoma or primary angle-closure glaucoma of 126 subjects were included.

For univariate and multivariate logistic regression analyses, the eyes were excluded for which reliable fundus planimetric results were unobtainable or in which cataract surgery had been performed, because magnification correction factor for fundus images is calculated using the parametric values of Gullstrand’s schematic eye^46,47^ and these parametric values are not established for such eyes; glaucomatous eyes with unreliable HFA results (fixation loss > 33% and/or false-positive results > 20%) also were excluded, because the mean deviation (MD) values were included as an explanatory variable in glaucomatous eyes. Eyes with secondary glaucoma also were excluded, because most had undergone a cataract surgery or had media opacities. Finally, 3638 normal eyes (3354 and 284 eyes with normal and abnormal or FP FDT results, respectively) of 2255 subjects and 153 eyes with primary open-angle glaucoma (POAG) or primary angle-closure glaucoma (PACG) (73 and 80 eyes with normal or negative and abnormal or FN FDT results, respectively) of 126 subjects were included to determine the factors associated with FP and FN responses to the FDT C-20-1 screening program in ophthalmologically normal and glaucomatous eyes, respectively (Fig. 1 and Table 4).

**Table 4:** Demographic data from 3638 normal eyes of 2255 normal subjects and 153 eyes with definite glaucoma of 126 patients with glaucoma for whom all parameters values used for analysis were available.

	Normal	Glaucoma
No. eyes (subjects)	3638 (2255)	153 (126)
Age (years)	56.9 (12.1)	67.6 (11.7)
Male/female	1840/1798	84/69
Presence/absence of diabetes	275/3363	16/137
Best-corrected visual acuity (logMAR)	− 0.04 (0.11)	0.03 (0.19)
Right/left	1693/1945	87/66
Intraocular pressure (mmHg)	14.7 (2.9)	16.5 (3.4)
Central corneal thickness (μm)	516 (33)	515 (34)
Spherical equivalent refraction (diopters)	0.04 (1.69)	0.24 (1.94)
Axial length (mm)	23.43 (0.90)	23.39 (0.85)
Anterior chamber depth (mm)	3.13 (0.38)	2.98 (0.40)
Disc area (mm^2^)	2.54 (0.50)	2.75 (0.57)
Rim area (mm^2^)	1.67 (0.30)	1.27 (0.34)
Vertical cup/disc ratio	0.56 (0.09)	0.73 (0.10)
β-peripapillary atrophy area (mm^2^)	0.44 (0.63)	0.95 (0.93)
Presence/absence of disc hemorrhage	6/3632	13/140
Mean deviation (decibels)	–	− 5.87 (6.43)

The data are expressed as the mean (standard deviation).

*logMAR* logarithm of the minimum angle of resolution.

### Data analysis

All information was stored to protect the participants’ privacy at the Data Analysis Center of the Ryukyu University Faculty of Medicine. The code number for each participant was stored separately from all examination data in a Kumejima municipal office. The data were double-checked and validated through inspection. Factors related to the positive responses in ophthalmologically normal eyes (FP responses) and those to the negative responses in glaucoma eyes (FN responses) were analyzed using SAS version 9.4 software (SAS Institute Inc., Cary, NC). The explanatory variables for an exploratory purpose were presence/absence of diabetes, laterality, refractive errors, presence/absence of cataract, presence/absence of disc hemorrhages, CCT, IOP, vC/D) ratio, disc, rim and β-peripapillary area, and the MD value of HFA 24–2 and the glaucoma type (the more pressure-dependent group, i.e., eyes with POAG with elevated pressure exceeding 21 mmHg or with PACG; and eyes with POAG with normal pressure of 21 mmHg or less) in cases of glaucoma. The univariate generalized linear mixed-modeled logistic regression analysis was performed and then variables with a *P* ≤ 0.10 were included in multivariate generalized linear mixed-modeled logistic regression analysis to adjust for the confounding effects of multiple possibly associated factors. *P* < 0.05 was considered significant.

## Data Availability

The data are from the Kumejima Study and are owned by Japan Glaucoma Society (JGS). Based on the agreement between the ethical committee of Kumejima Town and JGS concluded in May, 2005 and the municipal law of Kumejima Town for protecting private information, access to the original data is restricted to researchers who are members of JGS and are accepted by the society. To manage the epidemiological data which JGS gathered, JGS has the Epidemiology Study Group Data Center, which can be contacted at: epid.jgs@nifty.com. Upon reasonable and official request to the Data Center, anonymized data will be shared with the requester.
